# Protective Effect of Pogostone on 2,4,6-Trinitrobenzenesulfonic Acid-Induced Experimental Colitis via Inhibition of T Helper Cell

**DOI:** 10.3389/fphar.2017.00829

**Published:** 2017-11-17

**Authors:** Jiyan Su, Cailan Li, Xiuting Yu, Guanghua Yang, Jianhua Deng, Ziren Su, Huifang Zeng, Jiannan Chen, Xiaojun Zhang, Xiaoping Lai

**Affiliations:** ^1^State Key Laboratory of Applied Microbiology Southern China, Guangdong Provincial Key Laboratory of Microbial Culture Collection and Application, Guangdong Institute of Microbiology (CAS), Guangzhou, China; ^2^Guangdong Provincial Key Laboratory of New Drug Development and Research of Chinese Medicine, School of Chinese Materia Medica, Guangzhou University of Chinese Medicine, Guangzhou, China; ^3^The First Affiliated Hospital of Chinese Medicine, Guangzhou University of Chinese Medicine, Guangzhou, China; ^4^International Institute for Translational Chinese Medicine, Guangzhou University of Chinese Medicine, Guangzhou, China; ^5^Institute of Higher Education, Guangzhou University of Chinese Medicine, Guangzhou, China

**Keywords:** pogostone, TNBS, experimental colitis, anti-inflammation, T helper cell

## Abstract

Inflammatory bowel disease (IBD) is a chronic immune-related disease mainly caused by the disequilibrium of T helper (Th) cell paradigm? Pogostone (PO) is one of the major chemical constituents of *Pogostemon cablin* (Blanco) Benth. The present study aims to investigate the potential benefit of PO against IBD in a 2,4,6-trinitrobenzenesulfonic acid (TNBS)-induced experimental colitis model. PO treatment by enema significantly brought down the disease activity index (DAI) of the TNBS-challenged rats, which was manifested by the ameliorated inflammatory features including ulceration, adhesion, and edema. Hematoxylin-eosin (HE) staining and immunohistochemistry analysis showed that PO effectively relived colon damage by restoring epithelium, and more importantly, by inhibiting the infiltration of pro-inflammatory Th1 and Th17 cells in the colon. Additionally, PO inhibited the activity of myeloperoxidase and secretion of inflammatory cytokines including IFN-γ, IL-12p70, IL-17A, and IL-10. Together with our previous findings, the present data indicated that the anti-IBD effect of PO probably related to its direct inhibition on Th cell proliferation and suppression of the cytokines secretion. These results highlighted the potential of PO as a promising candidate to relieve IBD.

## Introduction

Inflammatory bowel disease (IBD) is a chronic immune-related disease that mainly involves the colon. There are two main entities of IBD, the ulcerative colitis (UC) and the Crohn’s disease (CD), depending on their specific pathological characteristics in terms of intestinal distribution, histological appearance, and some clinical complications (de Souza et al., 2017). Incidence and prevalence of IBD may have stabilized in high-incidence areas such as North America and Europe, but they keep rising in previously low-incidence areas such as Eastern Europe, Asia, and much of the developing world, which is especially high in the young adults (Molodecky et al., 2012; Ng, 2014; Kaplan and Ng, 2017). Therefore, World IBD Day (2017) is marked on 19 May each year since 2012, leading by patient organizations representing 35 countries on four continents, for global attention and support^1^. Most of the current remedies have been designed to directly control or remiss the inflammatory response of IBD, which is mainly a combination of anti-inflammatory agents (such as sulfasalazine and mesalazine), glucocorticosteroids, immunomodulating drugs (including azathioprine, methotrexate, and cyclosporine), and more recently the biological neutralizer (like infliximab, adalimumab, and certolizumab). Nevertheless, physicians and researchers keep seeking resolutions for IBD due to the unsatisfied efficacy and adverse drug reactions, such as parkinsonism, gastrointestinal, and liver adverse effects (Rogler, 2010; Yilmaz et al., 2014; Henriksen and Eriksson, 2016).

Although the etiology of IBD is still unclear, the disorder in the gut immune function, which was first proposed by Sartor in 1997 (Sartor, 1997), has been verified as the crux of IBD. That is, the disequilibrium of T helper cell (Th) subsets, as well as the consequent over reaction by any of them, contributes to the complexity of IBD immunopathogenesis. While CD is predominantly characterized by an enhanced aggregation and activation of type 1 T helper cell (Th1), UC has, however, been referred to a non-conventional Th2 response, and each has a unique cytokine profile that is essential for its unique condition (Coskun et al., 2016; de Souza and Fiocchi, 2016). On the other hand, the focus of attention regarding T cell subsets in IBD has shifted from the classical Th1/Th2 paradigm to that of Th17/regulatory T cell (Treg) in the last 15 years. Increasingly evidence has prompted that plasticity of interleukin (IL)-17 secreting T helper (Th17) cells plays a crucial role in both conditions of IBD, of which the flexibility allow this subset to embrace pro-inflammatory and protective roles in mucosal immunity by secreting a spectrum of cytokines without requiring *de novo* differentiation of naïve T cells (Koenen et al., 2008; Harbour et al., 2015; Ueno et al., 2015). Collectively, even though many other cell types of the innate and adaptive immune system are indispensable for the interception of the immune response in IBD, T cells are the key coordinators as they integrate and orchestrate signaling and functions of other cells.

Nowadays, more and more endeavor has been focused on the effective natural product from the wisdoms of traditional medicines in IBD studies. In traditional Chinese medicine, *Pogostemon cablin* (Blanco) Benth. is a valuable medicinal and aromatic plant with thousands-of-years history for the treatment of sunstroke-related headache, fever, emesis, and even coma. Numerous experimental studies has proved that the efficacies of *P. cablin* include anti-inflammation and analgesia (Lu et al., 2011), immunoregulation (Qi et al., 2009), anti-emesis (Yang et al., 1999), and so on. Pogostone (PO), namely 4-hydroxy-6-methyl-3-(4-methyl-pentanoyl)-2-pyrone (C_12_H_16_O_4_, CAS: 23800-56-8), is one of the major chemical constituents of *P. cablin*. It has been revealed that PO is responsible for most activities of *P. cablin*, including those of antibacterial (Li et al., 2012) and anti-fungal (Yi et al., 2013). Given that PO was proved to be capable of directly blocking T cell proliferation and altering inflammatory cytokine profile (Su et al., 2015), it is rational to infer that PO would be the critical component accounting for the anti-IBD activity of *P. cablin*. Among animal models applied in the studies of IBD, 2,4,6-trinitrobenzenesulfonic acid (TNBS) induction is a widely used chemical approach for its reproducibility and ease of development. Thus, in the present study, a TNBS-induced experimental colitis model was employed to investigate the potential benefit of PO against IBD.

## Materials and Methods

### Animals

Thirty-six Sprague-Dawley rats (male, 200 to 220 g) were provided by Laboratory Animal Center of Guangzhou University of Chinese Medicine (GZUCM, Guangzhou, China) All animals were housed at 20 ± 2°C with a humidity of 50% ± 5% in a 12 h light/dark cycle with food and water *ad libitum*. The animals were acclimatized for 7 d prior the experiment. The handling of animals and all experimental procedures performed were approved by the Committee for Animal Care and Use at GZUCM and in accordance with the Guide for the Care and Use of Laboratory Animals.

### Materials

Pogostone (PO, purity ≥ 98%) was isolated from the aerial parts of *P. cablin* as described previously (Li et al., 2012). *P. cablin* was authenticated by one of the authors (Pro. Xiaoping Lai) at the School of Chinese Materia Medica, Guangzhou University of Chinese Medicine, where a voucher specimen (No. 101009) was deposited. Chemical structure and purity of the obtained PO were identified and determined as reported previously (Yang and Xie, 1977; Chen et al., 2011).

2,4,6-Trinitrobenzenesulfonic acid, cetyltriethyl ammonium bromide, 3,3′-dianisidine, were purchased from Sigma (United States). Chloral hydrate was purchased from Daomao Chemical reagent factory (Tianjin, China). Salicylazosulfapyridine (SASP) was purchased from Shanghai pharmaceutical Co. Ltd. (Shanghai, China). Rat ProcartaPlex^TM^ Simplex Bead Set Reagent Kits was provided by eBioscience (San Diego, CA, United States).

### Induction of Colitis and PO Administration

Colitis was induced in accordance with the method as described previously (Scheiffele and Fuss, 2002). Briefly, rats were fasted for 18–24 h with free access to water. Rats were randomly assigned into control and colitis groups. For colitis groups, a total of 30 rats were anesthetized by 7% chloral hydrate (0.3 mL/kg, i.p.), and then subjected to a slow instillation of TNBS (25 mg/kg, 50% ethanol solution) into colon with a 6F medical grade catheter (8 cm proximal to the anus). A head down position was kept for 1 min to ensure TNBS remain in the colon. Another six rats, as Normal group, received equal volume of 50% ethanol alone by the same procedure. All animals were then given free access to food and water.

The TNBS-challenged rats were divided in to Model group (*n* = 6), SASP group (the positive control group, *n* = 6) and three PO groups (*n* = 6 in each group). From the day of colitis induction, rats of PO groups were administered with different doses of PO by enema, once a day (20, 40, and 80 mg/kg) for 7 days. PO was suspended in 5% sodium carboxymethylcellulose (CMC-Na). SASP was used as a positive control at a dose of 200 mg/kg daily by enema as well. Equal volume of solvent was given to both the Normal and Model groups. Throughout the experiments, rats were monitored for body weight loss. Twenty-four hours after the last PO administration, animals were sacrificed, and the colons were removed, dissected, and opened lengthwise for further analysis.

### Evaluation of Disease Activity Index (DAI)

According to previously established scoring system (**Table 1**), the DAI was evaluated as sum of the grade scores of weight loss, stool consistency, and occult/gross bleeding (Alex et al., 2009).

**Table 1:** Disease activity index (DAI) criteria.

Grade	Weight loss/%	Stool consistency	Occult/gross bleeding
0	0	Normal	N/A
1	1–5	Mild soft	–
2	5–10	Soft and wet	Hemoccult positive
3	10–20	Half loose stool	–
4	>20	Loose stool	Gross bleeding


### Assessment of Colonic Damage

Distal colon from anus to cecum was dissected rapidly and measured for its length. After cecum being removed, the rest part was opened longitudinally along colonic mesentery, washed with pre-cold PBS and measured the thickness of ulceration. Macroscopic injuries was assessed according to the criteria described in **Table 2** by Liu et al. (2013). Part of the colon was subjected to hematoxylin-eosin (HE) staining. The stained sections were observed and photographed under a light microscope (with 400× magnification), from which colon mucosa damage index scoring was assessed in a blinded fashion as described in **Table 3**, according to “Organizing Committee for Group of Digestive Tract Pathology (Csp, Chinese Society of Pathology) and Inflammatory Bowel Disease Group (Csg, Chinese Society of Gastroenterology) (2014)”.

**Table 2:** Macroscopic injuries score criteria.

Parameters	Score	Features
Ulceration	0	Normal
	1	Focal hyperemia, no ulcers
	2	Ulceration without hyperemia or bowel wall thickening
	3	Two sites of ulceration and inflammation
	4	Three major sites of damage extending >1 cm along the length of the colon
	5–10	Damage extended to >2 cm along the length of the colon, increasing the score by one for each additional centimeter of damage
Adhesion	0	No
	1	Mild: colon could be easily separated from peripheral tissues
	1.5	Medium: part of colon could not be separated from peripheral tissues
	2	Severe: colon could be hardly separated from peripheral tissues
Thickness	X	Increasing the score by one for each additional millimeter of thickness


**Table 3:** Histological grading criteria.

Score	Lymphocyte	Crypt	Colon wall
	infiltration^∗^	aberrant	aberrant
1	10%	Few deepening and distortion	The structure of colon wall is integrated.
2	10–25%	Mild deepening and distortion	Colon wall thickening, no ulceration
3	25–50%	Medium deepening distortion	Vascular proliferation, colon wall thickening invading into muscular layer, no ulceration
4	>50%	Severe deepening distortion	Colon wall thickening invading into muscular layer with ulceration


*^∗^The lymphocyte infiltration was observed at 400× magnification.*

### Immunohistochemistry

Tissue sections for immunohistochemistry were prepared from formalin-fixed, paraffin-embedded colon tissue. Stains against CD4 (Biorbyt, orb4830, rabbit, polyclonal, 1/400), IFN-γ (Biorbyt, orb10878, rabbit, polyclonal, 1/400), IL-17A (Abcam, ab79056, rabbit, polyclonal, 1/400) and Foxp3 (Abcam, ab22510, rabbit, polyclonal, 1/400) were performed according to the kit protocol (KGOS60, KeyGEN, Nanjing, China). Briefly, the slides were deparaffinized. Antigen retrieval for IFN-γ and IL-17A were carried out by incubation in 10 mM sodium citrate buffer with 0.1% Tween 20 in 100°C water bath for 20 min, while those for CD4 and Foxp3 were performed by incubation in EDTA buffer (pH 8.0) via microwave heating. Endogenous peroxidase in the tissue was blocked by incubation with 3% H_2_O_2_ in dark for 30 min. Tissues were blocked with 3% normal non-immune serum, and then they were incubated with primary antibodies at 4°C overnight following by an incubation with HRP-conjugated secondary antibodies at room temperature for 30 min. Lastly, the sections were stained with DAB substrate and counterstained with hematoxylin. The mean ratios of integrated optical density (IOD) to positive area (IOD/pixel) were used for analyzing the results of immunohistochemistry by Image Pro Plus 6.0 software (Media Cybernetics, Silver Spring, MD, United States).

### Measurement of Colonic Myeloperoxidase (MPO) Activity

Neutrophil infiltration into the colon was assessed indirectly by measuring MPO activity. The MPO activity assay was performed as described previously (Krawisz et al., 1984). The cleaned colon tissue segment (∼100 mg) was cut in to pieces and homogenized in 250 μL 0.5% cetyltriethyl ammonium bromide (in 50 mM PBS, pH 6.0) at 4°C followed by three freeze–thaw cycles (10 min/10 min) and a 15 s ultrasonic extraction in ice. The homogenate was centrifuged at 18,000 × *g* for 20 min at 4°C. The MPO enzymatic reaction was assessed by mixing 20 μL supernatant with 180 μL reaction buffer (containing 0.167 g/L 3,3′-dianisidine and 0.005 g/L H_2_O_2_ in 50 mM PBS, pH 6.0), and immediately scanned at 460 nm for 2 min at time point of 0 s, 40 s, 80 s, 120 s. The MPO enzyme activity (U/g) = (ΔA460/11.3 × (g/0.2), in which ΔA460 = [(OD_120s_ - OD_40s_) - (OD_80s_ - OD_0s_), g means tissue weight.

### Determination of Cytokine Profile in the Colon Tissue

About 30 mg colon tissue was minced, homogenized in chilled PBS with proteinase inhibitor cocktail (Roche, United States) for 30 s, and centrifuged at 18,000 × *g* for 20 min at 4°C. Protein content in the supernatant was quantified with BCA protein kit (Kang wei shiji Co. Ltd., Beijing, China). TGF-β was measured with TGF-β ELISA kit (Beijing Chenglin Biology Co. Ltd., Beijing, China), and IL-17A, IL-10, IL-4, IL-12p70, IFN-γ were determined by ProcartaPlex^TM^ Simplex Bead Set Reagent Kits (eBioscience, United States) according to the manufacturer’s instruction.

### Statistical Analysis

Statistical analysis was performed by one-way analysis of variance (ANOVA) using SPSS 18.0. A *post hoc* LSD test was applied for difference analysis under homogeneity of variance, if not, a Dunnett’s test would be applied. ^#^*p* < 0.05 and ^##^*p* < 0.01 as compared to Normal group; ^∗^*p* < 0.05 and ^∗∗^*p* < 0.01 as compared to Model group.

## Results

### PO Brought Down the DAI of Rats Suffering from TNBS-Induced Colitis

**FIGURE 1:**
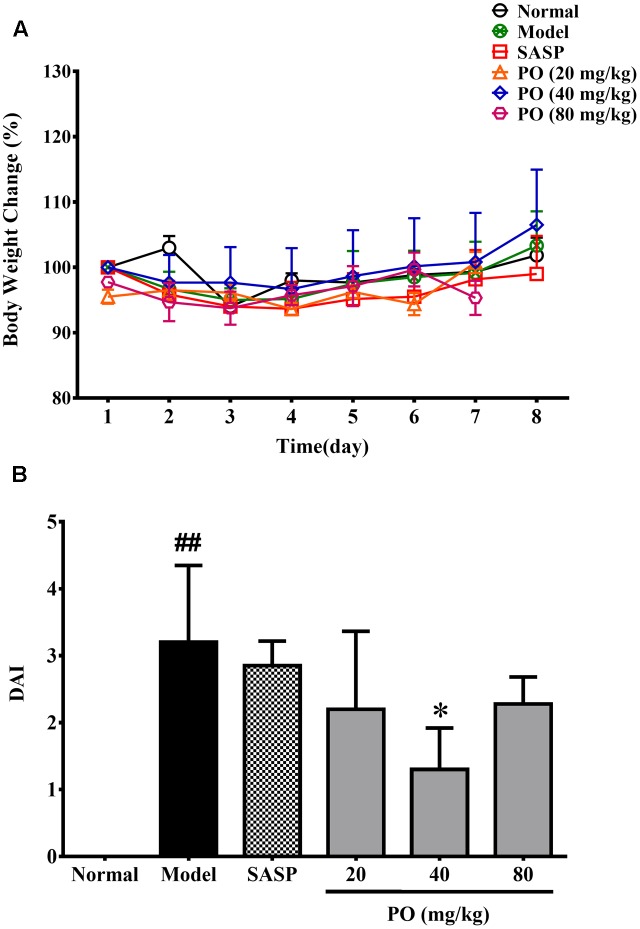
Body weight changes **(A)** and DAI assessment **(B)**. Values were represented the means ± SD (*n* = 6). ^#^*p* < 0.05 and ^##^*p* < 0.01 versus Normal group, ^∗^*p* < 0.05 and ^∗∗^*p* < 0.01 versus Model group.

As showed in **Figure 1A**, all the TNBS-challenged rats did not exhibited evident body weight loss compared with their normal counterparts. However, after the TNBS challenge, model group developed significant colitis signs (**Figure 1B**), which were characterized by lack of vitality, shaggy hair, loose and bloody stools, and a highest DAI scoring at 3.2 ± 1.1 (*p* < 0.01). By contrast, PO treatment reanimated the suffering rats, and turned their stools to be mild soft but less bloody. DAI were also brought down by PO treatment, scoring at 2.2 ± 1.2 (20 mg/kg), 1.3 ± 0.62 (40 mg/kg, *p* < 0.05), 2.3 ± 0.4 (80 mg/kg), respectively, suggesting that PO, especially the 40 mg/kg dosage, relieved TNBS-induced colitis.

### PO Ameliorated IBD Associated Colonic Damage

**FIGURE 2:**
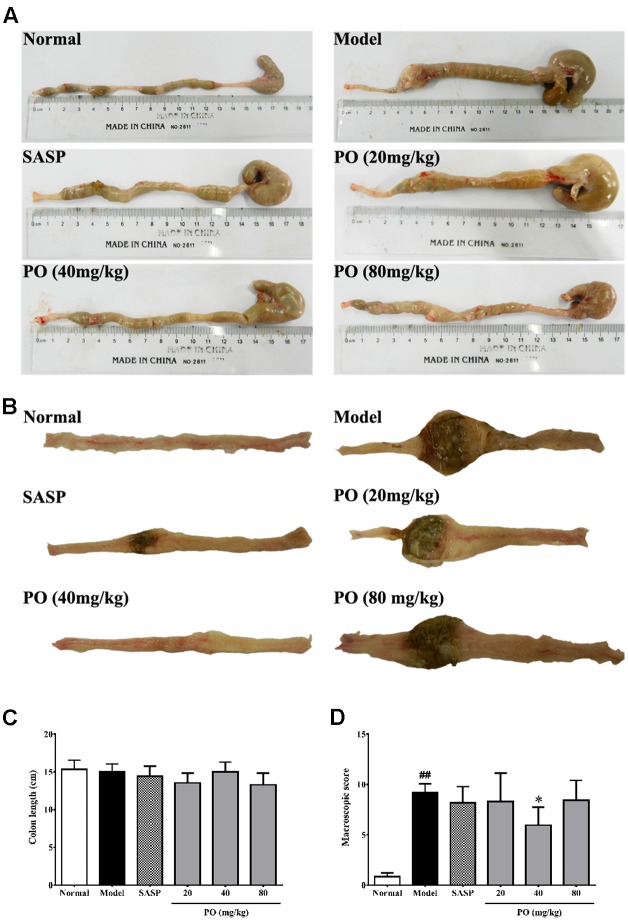
Macroscopical assessment for the colons. **(A)** Pictures of the whole colon (from anus to cecum). **(B)** Typical picture of the whole colon by longitudinal dissection. **(C)** Length of the colon. **(D)** Macroscopic score (MS) for colon injuries. Values were represented the means ± SD (*n* = 6). ^#^*p* < 0.05 and ^##^*p* < 0.01 versus Normal group, ^∗^*p* < 0.05 and ^∗∗^*p* < 0.01 versus Model group.

Macroscopical assessment including colon length (from anus to cecum) and injuries scoring was employed to assess the protection of PO against colonic damage. As indicated by the results (**Figures 2A,C**), TNBS challenge did not affect the colon length of the suffering rats. However, colons from the Model groups exhibited apparent inflammatory features, such as ulceration, severe adhesion, and thickening, which scored up to 9.2 ± 0.86 (**Figures 2B,D**, *p* < 0.01). As a clinical immunosuppressant, SASP (200 mg/kg) significantly relieved ulceration and colon wall edema, but did not eliminate adhesion, resulting in a score of 8.2 ± 1.6 (**Figures 2B,D**). PO (40 mg/kg), corresponding to the results of DAI evaluation, remarkably ameliorated the aforementioned inflammation characteristics, including adhesion, ulceration, and edema, which scored at 5.9 ± 1.8 (**Figures 2B,D**, *p* < 0.05 vs. Model group). Since both results from DAI assessment and macroscopical assessment indicated that 40 mg/kg PO exhibited the biggest benefit against TNBS-induced colitis, samples from the 40 mg/kg PO group were used for the further studies.

**FIGURE 3:**
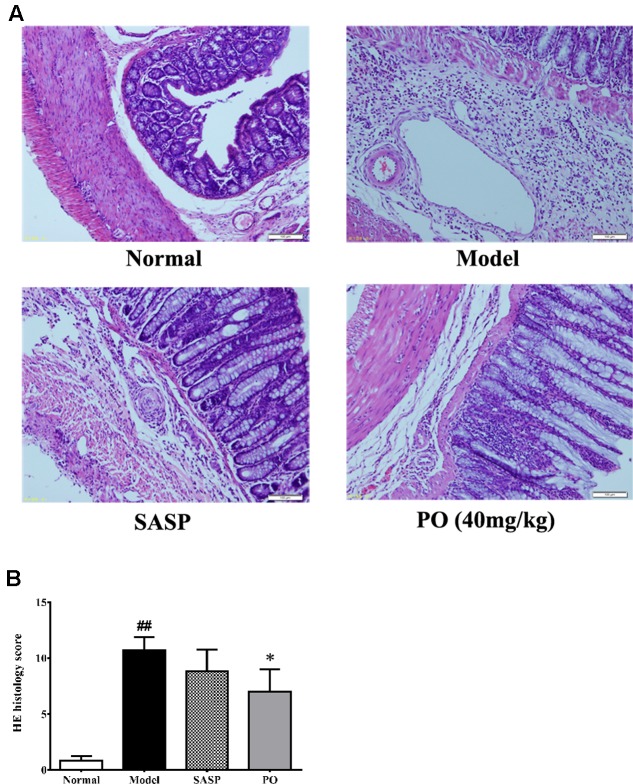
Histological evaluation for inflammatory lesion in colon tissue. **(A)** Representative HE staining picture for colon from each group (400× magnification). **(B)** Histology scores for tissue injuries from each group. Values were represented the means ± SD (*n* = 6). ^#^*p* < 0.05 and ^##^*p* < 0.01 versus Normal group, ^∗^*p* < 0.05 and ^∗∗^*p* < 0.01 versus Model group.

Hematoxylin-eosin staining was applied to provide more details about the effect of PO. As revealed in **Figure 3**, colons of Model group displayed significant structure lesion, such as shedding of epithelium, loss of goblet cell, inflammatory infiltration, and vessel proliferation, scoring extremely higher at 10 ± 1.21 (*p* < 0.01). By contrast, the score of SASP group was 8.8 ± 1.9, of which the colon exhibited less epithelium shedding, ulceration and leukocyte infiltration. Regarding to PO treatment (40 mg/kg), it was found that, in addition to the remission in epithelium, colons from the PO group showed much better crypt form and fewer vessel proliferations (7.0 ± 2.0, *p* < 0.05), suggesting that the colitis-induced colon lesion was significantly ameliorated by PO.

### PO Ameliorated Colon Damage by Inhibiting Pro-inflammatory Th1 and Th17 Cell Infiltration

**FIGURE 4:**
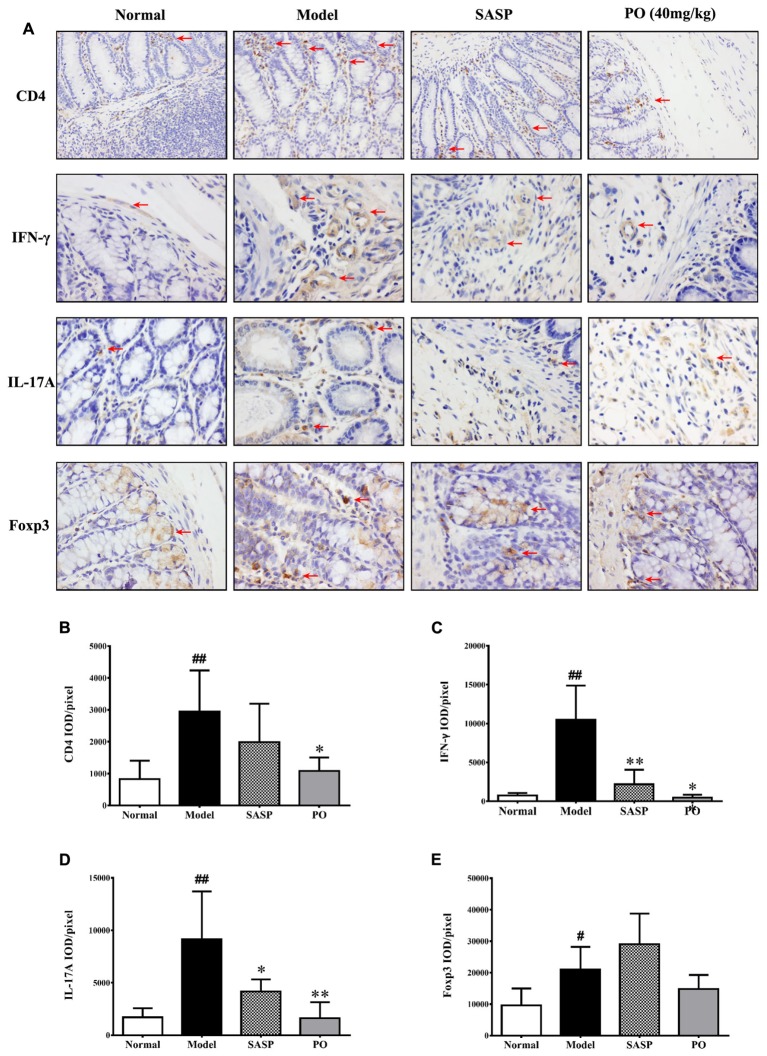
Immunohistochemistry detection for related proteins of Th cells. **(A)** Representative IHC picture (400× magnification) for CD4 (Th), IFN-γ (Th1), IL-17A (Th17), and Foxp3 (Treg), the positive area was indicated by red arrows. **(B–E)** The mean ratios of integrated optical density (IOD) to area (IOD/pixel) were used for analyzing the results of immunohistochemistry. Values were represented the means ± SD (*n* = 6). ^#^*p* < 0.05 and ^##^*p* < 0.01 versus Normal group, ^∗^*p* < 0.05 and ^∗∗^*p* < 0.01 versus Model group.

Given that Th cell plays a cardinal but dual role in the IBD development, we employed immunohistochemistry to investigate the Th cell infiltration in the inflammatory colon tissue. It was found that large number of Th cell (CD4^+^) existed in the TNBS-challenged rats (**Figure 4A**, the first row), as indicated by the significant higher ratio of IOD to positive area (**Figure 4B**, *p* < 0.01). Both SASP and PO inhibited Th cell infiltration, and PO especially made a remarkable reduction (*p* < 0.01).

Among the Th cell subsets, Th1 (IFN-γ^+^) and Th17 (IL-17A^+^) are the pro-inflammatory ones that drive the development of IBD, while regulatory T cell (Treg, Foxp3^+^) is the one that suppresses the inflammation, thus it is necessary to make a more detailed observation for their infiltration in the colon. In accordance with the increase of total Th cell, Th1, Th17, and Treg cells obviously increased in the colon tissue of Model group with higher ratios of IOD to positive area (**Figure 4A**, from the second row to the fourth row), and those of Th1 (IFN-γ^+^) and Th17 (IL-17A^+^) increased much more than that of Treg (**Figures 4C–E**, *p* < 0.01). Interestingly, both SASP and PO evidently suppressed the excess infiltrations of pro-inflammatory Th1 and Th17 (*p* < 0.01), but did not apparently affected the distribution of Treg cell.

### PO Suppressed Myeloperoxidase Activity and Inflammatory Cytokine Secretion

**FIGURE 5:**
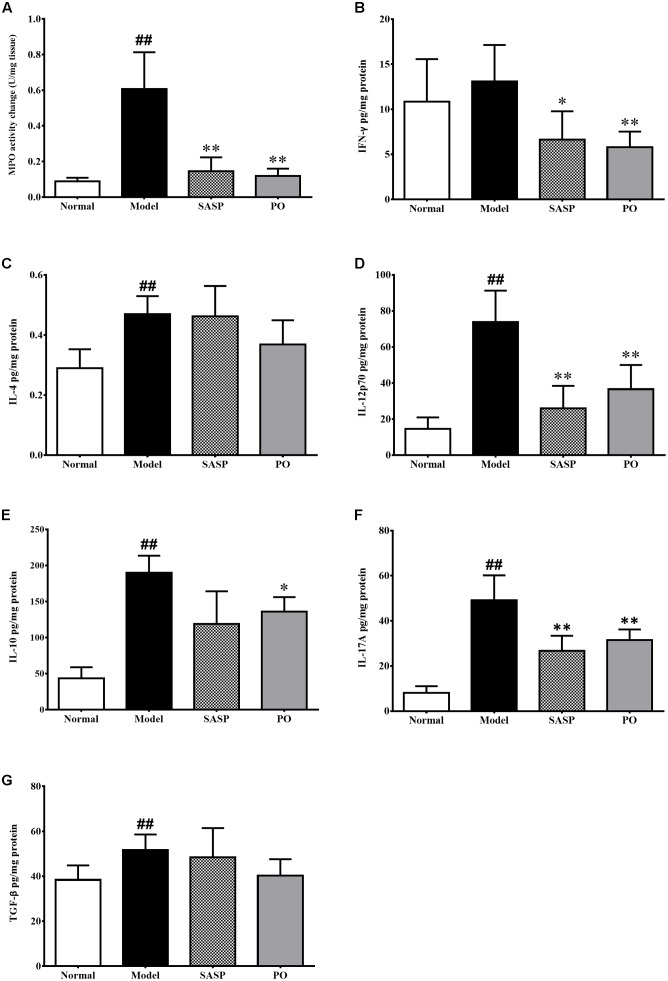
MPO activity **(A)** and cytokine profiling **(B–G)** in the colon tissue. Values were represented the means ± SD (*n* = 6). ^#^*p* < 0.05 and ^##^*p* < 0.01 versus Normal group, ^∗^*p* < 0.05 and ^∗∗^*p* < 0.01 versus Model group.

Myeloperoxidase (MPO) is a heme protease that is secreted by neutrophile granulocyte, monocyte and macrophage, and serves as a marker of neutrophile granulocyte activity. As showed in **Figure 5A**, MPO activity of the Model group (0.61 ± 0.21 U/mg tissue) were extremely higher than that of normal group (0.088 ± 0.021 U/mg tissue, *p* < 0.01). By contrast, those of SASP and PO (40 mg/kg) groups were remarkably decreased to 0.15 ± 0.077 U/mg tissue (*p* < 0.05) and 0.12 ± 0.040 U/mg tissue (*p* < 0.01).

Changes of the inflammatory cytokines profile in the colon revealed more details about the activity of PO. As showed in **Figures 5B–G**, TNBS challenge strikingly elevated the levels of IL-4, IL-12p70, IL-10, IL-17A, and TGF-β (*p* < 0.01). In comparison, both SASP and PO remarkably suppressed most of them. In brief, SASP made significant suppressions on IFN-γ, IL-12p70, and IL-17A, while PO decreased the pro-inflammatory cytokines including IFN-γ, IL-12p70, IL-17A, as well as the anti-inflammatory IL-10, which was interestingly quite accordance with the depression on Th cell infiltration. Nevertheless, TGF-β was not affected by both reagents. Together with the results from the macroscopical and microscopical assessments, it can be speculated that the anti-inflammatory effect of PO on IBD possibly attributes to suppression on the Th-cell immune response.

## Discussion

Inflammatory bowel disease is characterized by an increased infiltration of leukocytes into the inflamed intestinal mucosa, thus resulting in a persistent inflammation. Pathologically, the mucosa injuries caused by leukocytes infiltration include ulceration, necrosis, edema, and even blooding, which not only impair the nutrients intake and metabolism in the guts, but also induce abnormalities in peristalsis and submucosal enteric nervous system. Therefore, typical signs of IBD always consist of weight loss, abdomen pain with diarrhea, fecal occult blood, and bloody stools, which have been employed as criteria for assessment in clinical and in animal researches (Wolf et al., 2012; Walsh et al., 2016). TNBS is a classical inducer in IBD animal model. Along with the damage on intestinal mucosal barrier by alcohol, TNBS would permeate into the mucosa where it turns to be a holoantigen by conjugated with macromoleculars, finally elicits induces transmural inflammation in the gut with morphological and histopathological features similar to those of human IBD (Randhawa et al., 2014), which is, especially, attributed to the predominant activation of Th-mediated immune response. As shown in the results, PO exhibited a promising therapeutical effect by relieving the TNBS-induced DAI. Moreover, the amelioration by PO was confirmed by the alleviated inflammatory colon injuries, including less ulceration or leukocyte infiltration, reduced colon edema, and eliminated adhesion, and more detailed by the remission of epithelium lesion, improved crypt form and fewer vascular proliferations.

As afore-mentioned, leukocyte trafficking plays a central role in the immunopathogenesis of IBD, and inhibition of leukocyte trafficking to the gut mucosa has been recognized as an important target for the development of IBD drugs (Danese and Panés, 2014). MPO is one of the inflammation indicators for IBD, which is mainly produced by many kinds of leukocytes, such as neutrophile granulocyte, monocyte, and macrophage to eliminate microbes or pathogens. However, MPO would induce topical oxidative stress and tissue damage once it generates excess amounts of oxidants like hypochlorous acid and tyrosyl radical (Shao et al., 2010). As showed in our results, PO (40 mg/kg) remarkably suppressed the activity of MPO in the colon, suggesting that PO brought about an effective alleviation on the inflammation status, thereby restoring the self-injured tissue.

Among the offensive leukocyte giving rise to IBD, Th cells are the most concerned factors that contribute to its complexity (de Souza and Fiocchi, 2016). According to the specific secretory cytokines and transcription factors, proliferating Th cells that develop into effector T cells would differentiate into several categories. Th1 cell (IFN-γ^+^ as symbol) is an effector Th cell that is characterized by secretion of IFN-γ, IL-2 and IL-12 to mediate cytotoxic immune response involved in cellular immunity and hypersensitivity; while Th2 (IL-4^+^ as symbol) features in secreting IL-4, IL-5, and IL-6 to promote B cell and antibody production for humoral immunity. Accumulating clinical studies has pointed that the imbalance of Th1/Th2 paradigm are important in chronic inflammatory process of IBD (Sartor, 1997; Abraham and Cho, 2009; de Souza and Fiocchi, 2016), and it may be further associated with disease activity and the response to tumor necrosis factor inhibitor in patients with IBD (Li et al., 2016). Th17 (IL-17^+^ as symbol) has been heavily implicated in tissue-specific immune pathology not only in the murine models of IBD, but also in human CD and UC (Ueno et al., 2015). Treg, on the other hand, counters the proliferation and activation of effector T cells via suppressing cytokines such as IL-10 and TGF-β, so as to maintain tolerance to self-antigens, and prevent autoimmune disease (Saruta et al., 2007). Data in the present study showed that PO decreased the infiltration of total Th cells in the inflammatory colon, especially those of pro-inflammatory Th1 (IFN-γ^+^) and Th17 (IL-17A^+^) but not that of Treg. These results indicate that inhibition on Th cell contributes to the protective effect of PO against TNBS-induced colon injury.

In addition to Th cell infiltration, disorder of cytokine profile is another pivotal indicator of the Th cell-mediated overreaction in IBD. Cytokines are critical proteins secreted by lymphocytes through autocrine or paracrine, for the purpose of function execution and signaling transition. They can be classified as the pro-inflammatory ones that enhance the activities of lymphocytes to boost or to drive inflammation, and their anti-inflammatory counterparts that negatively regulate these activities. Hence, to orchestrate the cytokines profiling, such as Infliximab targeting TNF-α blockage, has been a novel and effective strategy to control the severity of IBD, though it always accompanies with multiple side effects (Shah et al., 2014; Yilmaz et al., 2014; Henriksen and Eriksson, 2016). IL-4, IFN-γ, IL-12p70, and IL-17A are considered as pro-inflammatory cytokines that are produced excessively and chronically and they contribute greatly to the inflammatory reaction in IBD. TGF-β and IL-10, on the opposite, usually act as anti-inflammatory cytokines to inhibit the proliferation and functions of T cells via downregulating expressions of cytokines, MHC class II antigens, and co-stimulatory molecules (Sanjabi et al., 2017; Szondy et al., 2017). In this study, PO suppressed both pro-inflammatory cytokines (IFN-γ, IL-12p70, IL-17A) and the anti-inflammatory one (IL-10), suggesting an inhibition on the hyperactive immune response. Together with all the above findings, it is plausible that PO restrains the Th cell infiltration and poses a comprehensive inhibition on inflammatory cytokines profile, and finally remiss the inflammation in IBD.

Collectively, our data revealed that PO as an protect animal against experimental IBD via inhibiting the infiltration of pro-inflammatory Th1 and Th17 cells, suppressing MPO activity and decreasing inflammatory cytokines, i.e., IFN-γ, IL-12p70, IL-17A, and IL-10. Nevertheless, PO did not affected mRNA expression of the specific transcript factor for Th1 (*T-bet* and *Stat1*), Th2 (*Gata3* and *Stat6*), Th17 (*Ror-gama t* and *Stat3*), and Treg (*Foxp3*) (Data was provided in the Supplementary Material), suggesting that the restrains of PO on Th1 or Th17 cell infiltration had no concern with the regulation on Th cell differentiation into specific functional subset. In our previous study, PO was demonstrated to be able to directly block T cell proliferation via down-regulation of cyclin E, cyclin B, and CDK1 and the subsequent S-phase arrest without toxicity on the cell vitality, exhibiting a blunt on the T cell-associated immune response (Su et al., 2015). Given that either entity of IBD, UC, or CD, is characterized by its own Th cells function and cytokine profile (Coskun et al., 2016; de Souza and Fiocchi, 2016), our findings indicated that the underlying mechanism of anti-IBD effect of PO would probably rely on its direct blockade on the proliferation of the whole Th cell population, following by a suppression on the cytokines secretion, thereby relieving the inflammatory response induced by TNBS/alcohol. Additionally, our previous work demonstrated that PO was endowed with a considerable drug safety both *in vitro* and *in vivo* (Li et al., 2012; Su et al., 2015), highlighting the potential of PO as a promising candidate for the treatment of IBD. Therefore, ongoing work will focus on the comprehensive understanding about how PO orchestrates the cell proliferation mechanism, such as whether the epigenetic changes of T cell is induced and what kinds of key signaling pathways are involved in (e.g., MAPK, PI3K/Akt, and mTOR), so as to understand the therapeutic mechanism of PO.

## Author Contributions

Conceived and designed the experiments: XZ and XL. Performed the experiments: JS, CL, XY, GY, and JD. Analyzed the data: JS, CL, ZS, HZ, and JC. Drafted and revised the manuscript: JS, CL, XZ, and XL.

## Conflict of Interest Statement

The authors declare that the research was conducted in the absence of any commercial or financial relationships that could be construed as a potential conflict of interest.

## Footnotes

1 https://worldibdday.org/world-ibd-day-events/ibd-day-2012
