# Supplementary material for: Protective Effect of Pogostone on 2,4,6-Trinitrobenzenesulfonic Acid-Induced Experimental Colitis via Inhibition of T Helper Cell
Source: Front Pharmacol. 2017 Nov 17;8:829. doi: 10.3389/fphar.2017.00829 (PMC5699238; doi:10.3389/fphar.2017.00829)
Supplement: Supplementary file 1 [file Data_Sheet_1.docx]

**Analysis of transcript factors mRNAs by Real-time PCR**

Material and methods

Total RNA was extracted from colon tissues by using TRIzol according to the manufacturer’s instructions (Invitrogen, USA). 1μg total RNA was reverse transcribed using the GoScript reverse transcription system (ThermoScience, K1622, USA) following the supplier’s protocol. The reactions were incubated at 25℃ for 5 min, then at 42℃ for 60 min, and terminated at 70℃ for 5 min. The PCR primer sequences are listed in Table 1. Real-time PCR reactions were performed as follows: a precycling stage at 95℃ for 30 s, then 40 cycles of denaturization at 95◦C for 10 s and annealing at 60◦C for 30 s. Fluorescence was measured at the end of each annealing step, and the melting curves were monitored to confirm the specificity of the PCR products. The 2^−△△Ct^ method was used to determine the mRNA expression levels of mucin-1 and mucin-2relative to control gene β-actin, in which △△Ct=(Ct _target gene_-Ct _β-actin_)_Treat group_-(Ct _target gene_-Ct _β-actin_)_Normal group_ .

Table 1 Primers for real-time PCR

| Gene | | Primer | Product length (bp) |
| --- | --- | --- | --- |
| *Stat1* | Sense | CGAAGAGCGACCAGAAAC | 212 |
|  | Antisense | CTGATCCAGGCAGGCATT |  |
| *T-bet* | Sense | CCTGCTGGACGACAATGG | 170 |
|  | Antisense | TCTGGTAGGCGGTGACTG |  |
| *Stat6* | Sense | CAGCCTCTTGCAGCACAT | 211 |
|  | Antisense | CCTGGTCTCCCTTACTCG |  |
| *Gata3* | Sense | GCCAGGCAAGATGAGAAAG | 148 |
|  | Antisense | AGGGCGGATAGGTGGTAAT |  |
| *Ror-gama t* | Sense | CGCACCAACCTCTTCTCACG | 195 |
|  | Antisense | GACTTCCATTGCTCCTGCTTTC |  |
| *Stat3* | Sense | AAAGGACATCAGTGGCAAGA | 303 |
|  | Antisense | ATCGGCAGGTCAATGGTA |  |
| *Foxp3* | Sense | GCACAAGTGCTTTGTGCGAGT | 571 |
|  | Antisense | TGTCTGTGGTTGCAGACGTTGT |  |
| *β-actin* | Sense | ACGTTGACATCCGTAAAGAC | 203 |
|  | Antisense | CTGGAAGGTGGACAGTGAG |  |

Results





Figure 1 Real-time PCR for transcript factors mRNAs in the colon tissue. (A) *T-bet*; (B) *Stat1*; (C) *Gata3*; (D) *Stat6*; (E) *Ror-gama t*; (F) *Stat3*; (G) *Foxp3*. Values were represented the means ± SD (n= 6). # *p* < 0.05 and ## *p* < 0.01 versus Normal group, * *p* < 0.05 and ** *p* < 0.01 versus Model group.
